# Draft genome sequences of *Buchnera aphidicola* from three aphid species (Hemiptera: Aphididae: Eriosomatinae) associated with gall formation on elm trees

**DOI:** 10.1128/mra.00336-25

**Published:** 2025-06-20

**Authors:** Xin Tong, Yuuki Kobayashi, Mika Ikeda, Hsin-i Wen, Shin-ichi Akimoto, Shuji Shigenobu

**Affiliations:** 1 Laboratory of Evolutionary Genomics, National Institute for Basic Biology 68292, Okazaki, Aichi, Japan; 2 Department of Ecology and Systematics, Graduate School of Agriculture, Hokkaido University 12810, Sapporo, Japan; 3 Metabolome Informatics Research Team, RIKEN Center for Sustainable Resource Science 98319, Yokohama, Kanagawa, Japan; University of Maryland School of Medicine, Baltimore, USA

**Keywords:** *Buchnera aphidicola*, complex life cycles, gall-forming aphids

## Abstract

The *Buchnera aphidicola* genomes from eriosomatine gall-forming aphids *Tetraneura sorini*, *Tetraneura akinire*, and *Eriosoma harunire* were sequenced, with genome sizes of 533,871, 530,863, and 627,315 bp, respectively. These genomes shed light on *Buchnera*’s role in aphid symbiosis and adaptation.

## ANNOUNCEMENT

*Buchnera aphidicola* is an obligate nutritional endosymbiont of aphids (Hemiptera: Aphididae), providing essential amino acids for their survival and development (1–5). To facilitate studies on aphid–symbiont–plant interactions, we sequenced *Buchnera* genomes from three eriosomatine aphids that feed on the same Japanese elm species (*Ulmus davidiana* var. *japonica*) but induce distinct gall morphologies (6). These genomes offer a valuable resource for investigating functional variation in *Buchnera* across closely related hosts.

Mature gall colonies of *Tetraneura sorini* (TS), *Tetraneura akinire* (TA), and *Eriosoma harunire* (EH) were collected from Japanese elm trees in Sapporo, Japan, in June (43.0915°N, 141.4382°E for *T. sorini* and *T. akinire*) and July (43.0709°N, 141.3432°E for *E. harunire*) of 2020. Specimens were preserved in 99.99% ethanol at –20°C prior to DNA extraction. DNA from a single gall colony, including both aphid and *Buchnera* genomes, was extracted using the QIAGEN Genomic-tip 20/G without DNA shearing or size selection. Quality control of the extracted gDNA was conducted using a Qubit 2.0 Fluorometer and Nanodrop ND-2000C (Thermo Fisher Scientific).

Nanopore sequencing libraries were prepared using the SQK-LSK109 kit (Oxford Nanopore) without DNA fragmentation and sequenced on a GridION platform (FLO-MIN111). Basecalling was performed with Guppy v4.2.3 in high-accuracy mode (Q ≥ 7). Illumina libraries were prepared with the TruSeq RNA Library Prep Kit (average insert size ~470 bp) and sequenced on an Illumina NextSeq 550 platform in paired-end 75 bp mode. Adapter trimming was performed by the sequencing facility, and quality was assessed using FastQC v0.11.9 (7).

*De novo* assembly of Nanopore reads was performed by detecting overlaps with Minimap2 (8) and assembling with Miniasm (9). The yield circularized contigs were identified as *Buchnera* based on BLAST comparisons to reference genomes from *Acyrthosiphon pisum* (3) and *Baizongia pistaciae* (10). These assemblies were error-corrected using Racon (11), followed by polishing with Pilon v1.24 (12) for three times using quality-filtered Illumina reads. Default parameters were used unless otherwise specified. The finalized assemblies were then reoriented to align with the genomic structure of *Buchnera* from *Acyrthosiphon pisum* (3) for consistency. Genome annotation was conducted using Prokka v1.14.6 (13), and annotation files are publicly available on FigShare (14).

**TABLE 1:** Genomic features and NCBI accession numbers of *Buchnera aphidicola* from three eriosomatine aphids

Strain	Genome size (bp)	Number of contigs	Plasmid	Protein-coding genes	Pseudogenes	tRNA	rRNA	GC (%)	Depth (x)^a^	N50 (bp)	No. of reads (Illumina / Nanopore)	No. of reads (Illumina / Nanopore)	SRA no. (Illumina / Nanopore)	Genome accession no.
TS	533,871	1	0	485	32	32	3	23.4	42X	4,510	46.7 M / 2.17 M	46.7 M / 2.17 M	SRR33632137/SRR32628561	CP188087
TA	530,863	1	0	474	29	32	3	23.3	181X	3,570	48 M / 1.07 M	48 M / 1.07 M	SRR33632138/SRR32628560	CP188088
EH	627,315	1	0	489	17	32	3	23.7	431X	3,150	43.1 M / 1.82 M	43.1 M / 1.82 M	SRR33632139/SRR32628559	CP188086

^a^ The sequencing depth (x) was calculated using Nanopore sequencing reads.

## Data Availability

The accession numbers for Nanopore and Illumina SRA data, as well as genome assemblies generated in this study for the three *Buchnera aphidicola* strains isolated *T. sorini*, *T. akinire*, and *E. harunire*, are provided in Table 1 with links. The Prokka annotation files for the three *Buchnera aphidicola* strains are available at https://doi.org/10.6084/m9.figshare.29096333.v1.
